# Identification of Benthic Foraminifera Presence in The Marginal Environments of Biliran Island, Philippines

**DOI:** 10.21315/tlsr2024.35.3.14

**Published:** 2024-10-07

**Authors:** Ernil D. Sumayao, Andrew S. Dy

**Affiliations:** 1 School of Teacher Education, Biliran Province State University, Naval, Biliran, 6560 Philippines; 2 Department of Teacher Education, University of San Carlos, Cebu City, Cebu, 6000 Philippines

**Keywords:** Benthic Foraminifera, Marine Ecosystem, Marine Water Quality, Environmental Management

## Abstract

Benthic foraminifera are unicellular marine micro-organism with a hard exoskeleton and commonly present in the benthic community of marine ecosystem. This study aimed to identify the benthic foraminifera present along the coastal areas of eight municipalities in Biliran Island, Philippines. Quadrat sampling was conducted and three samples per quadrant transect of 1 m × 1 m divided into nine squares were collected. The samples were then observed under the microscope, and the specimens seen were identified by comparing them with the images of the sample species from the website https://marinespecies.org/. The researchers conducted an *in-situ* collection of the foraminiferal shells from intertidal areas along shallow water coastlines of the island. Results showed that the coastal environment of Biliran Island has the presence of the genera *Spirillina, Quinqueloculina, Marginopora* and *Sorites*. The identified species were classified based on their feeding mechanisms as herbivory and passive suspension feeding. The presence of benthic foraminifera species along the coastal environments of Biliran Island provides a record of the environment where they are found, making them natural bioindicators of water quality. This study provides a baseline for further studies on the distribution and abundance of benthic foraminifera in the area and can contribute to the understanding of the environmental conditions of the coastal areas in Biliran Island.

**Highlights**
- Benthic foraminifera hard exoskeletons are present in the coastal areas of Biliran Island, Philippines.
- The identified benthic foraminifera based on the exoskeletons collected are *Spirillina, Quinqueloculina, Marginopora* and *Sorites*.
- Presence of benthic foraminifera can be used as bioindicators of water quality.

## INTRODUCTION

Benthic foraminifera are single-celled marine micro-organisms with a hard exoskeleton made up of calcium carbonate or sediment particles (Saraswat & Nigam 2013). They are considered to be important bioindicators for monitoring marine and estuarine pollution in temperate regions and have been used as indicators for coral reef water quality in tropical, subtropical, and temperate environments (Alve 1995; Benito *et al*. 2015). Hallock (2000) proposed benthic foraminifera as indicators for coral reef water quality, and subsequent studies have confirmed their usefulness in this regard (Hallock *et al*. 2003). Benthic foraminifera have also been used to infer present and past environmental conditions of coastal ecosystems (Horton *et al*. 2007; Pruitt *et al*. 2010; Cheng *et al*. 2012; Takata *et al*. 2014), and to track environmental changes due to human impacts (Cearreta *et al*. 2002; Debenay & Fernandez 2009) and pollution from heavy metals (Alve 1995; Armynot du Chatelet *et al*. 2004; Li, Xiang *et al*. 2013; Li, Li *et al*. 2015).

Marine water quality is influenced by the presence of benthic foraminifera as several studies suggest. Alve (1995) reported that the abundance and diversity of benthic foraminifera in the sediment can provide information about the extent and severity of pollution in marine environments. The sensitivity of benthic foraminifera to environmental stressors such as pollution makes them valuable bioindicators for assessing water quality (Benito *et al*. 2015). Benthic foraminifera are important bioindicators for monitoring marine and estuarine pollution, and for assessing the quality of coral reef water and coastal ecosystems. Their sensitivity to environmental stressors such as pollution makes them useful in tracking environmental changes due to human impacts.

Benthic foraminifera have been recognised as excellent bioindicators for monitoring marine and estuarine pollution. Their presence, distribution, abundance and morphological abnormalities are sensitive indicators of the quality of the marine environment (Murray 2006). For example, benthic foraminifera’s diversity, richness and abundance have been found to decrease in polluted environments, indicating adverse environmental conditions (Korsun *et al*. 2013; de Jesus *et al*. 2020). In a study conducted by Schönfeld *et al*. (2011), benthic foraminifera were used as indicators to assess the impact of organic pollution on coastal ecosystems. They found that the abundance of several foraminiferal taxa, such as Elphidium, Ammonia and Bolivina, decreased significantly in polluted areas.

Furthermore, the presence of heavy metals in the marine environment has a significant impact on benthic foraminiferal assemblages. Studies have shown that heavy metal pollution affects the foraminiferal community structure, diversity, and abundance (Alve 1995; Armynot du Chatelet *et al*. 2004; Li, Xiang, *et al*. 2013; Li, Li, *et al*. 2015). For instance, Alve (1995) found that heavy metal pollution affected the species diversity of benthic foraminifera in the Oslofjord, Norway. Similarly, Armynot du Chatelet *et al*. (2004) observed a significant decrease in foraminiferal diversity and abundance in the vicinity of a zinc plant in the Bay of Seine, France.

In addition, benthic foraminifera have been used as indicators for tracking environmental changes due to human impacts such as dredging, land reclamation and coastal development (Cearreta *et al*. 2002; Debenay & Fernandez 2009). Moreover, the presence of benthic foraminifera in the marine environment has been found to be useful in tracing the present and past environmental conditions of coastal ecosystems (Horton *et al*. 2007; Pruitt *et al*. 2010; Cheng *et al*. 2012; Takata *et al*. 2014). Benthic foraminifera are excellent bioindicators for monitoring marine and estuarine pollution. Researchers have used benthic foraminifera as indicators to assess the impact of various pollutants. The use of benthic foraminifera as bioindicators has provided valuable insights into the present and past environmental conditions of coastal ecosystems. Therefore, the presence of benthic foraminifera can be used to assess the quality of marine water in different regions.

The island of Biliran’s natural resources are not only significant for tourism but also for the ecosystem. The quality of marine water in the province can impact its marine biodiversity, which affects the tourism industry and the livelihood of the people in the area. In a study conducted by Gomez *et al*. (1994), they found that the coral reefs of Biliran were highly diverse and had a high live coral cover. This indicates that the province has a healthy marine environment that needs to be maintained.

Furthermore, benthic foraminifera could serve as a bioindicator for the marine water quality in the area, as they have been used in previous studies to monitor pollution and changes in the environment. According to Frontalini and Coccioni (2011), the use of bioindicators has been an essential tool for the monitoring of the quality of water bodies. Benthic foraminifera, as mentioned earlier, are sensitive to changes in their environment, and their presence or absence could indicate the water quality in the province of Biliran. The assessment of the benthic foraminifera in the marine water of Biliran is crucial to the maintenance of the province’s ecosystem, tourism industry, and the livelihood of the people in the area. More studies on the island’s marine environment are necessary to understand the current status of the ecosystem and to develop effective management and conservation strategies.

## OBJECTIVE OF THE STUDY

This study aims to investigate the diversity of benthic foraminifera species along the coastal area of Biliran Island and to explore the ecological implications of their presence and absence. Specifically, the study aims to identify the benthic foraminifera species present in the area and analyse their potential ecological roles in the local marine ecosystem.

## METHODOLOGY

The relationship between the structure and composition of foraminifera assemblages and their environment has been extensively studied. Several studies have been conducted at single locations, and they have described this relationship (Ferraro & Hanauer 2014; El Albani *et al*. 2019; Mojtahid *et al*. 2009). Researchers have also used down-core benthic foraminifera assemblages to determine whether human activities have altered baseline conditions in marginal environments (Tsujimoto *et al*. 2018; Martinez-Colon *et al*. 2009; Alve *et al*. 2009; Dolven *et al*. 2013). The present study aimed to identify the benthic foraminifera species present along the coastal area of the Biliran Island and to determine the implications of the presence and absence of the benthic species. The researchers collected dead foraminiferal shells from intertidal areas along shallow water coastlines of Biliran Island. This study adds to the current knowledge of the relationship between foraminifera assemblages and their environment by providing information on the species composition of benthic foraminifera in the coastal area of Biliran Island.

In the study conducted by the researchers, a quadrat sampling method was employed to collect samples of dead foraminiferal shells along the shallow water coastlines of Biliran Island. The study sites were located in eight different municipalities, namely Naval, Almeria, Kawayan, Culaba, Caibiran, Cabucgayan, Biliran and Maripipi, to ensure a representative sample of the coastal areas. Within each study site, a quadrat measuring 1 m × 1 m was set up, and three samples were collected per quadrant transect. Each quadrat was divided into nine squares, and the researchers collected a small number of sand grains (sample) from each square.

The sand grains collected from each square were then placed on a microscope slide and observed under a microscope in the laboratory using a (4/0.1, 160/0.17) objective lens. The specimens seen under the microscope were photographed using a camera and compared with images of sample species from (https://marinespecies.org) for identification. The quadrat sampling method used in this study is a commonly employed technique in ecological studies to collect representative samples of a given area (Fernández-García *et al*. 2019; Padilla & Sutherland 2019). The use of a microscope and camera for identification is also a standard practice in benthic foraminifera studies (Holzmann *et al*. 2022; Wan *et al*. 2020).

## RESULTS AND DISCUSSION

**Figure 1:**
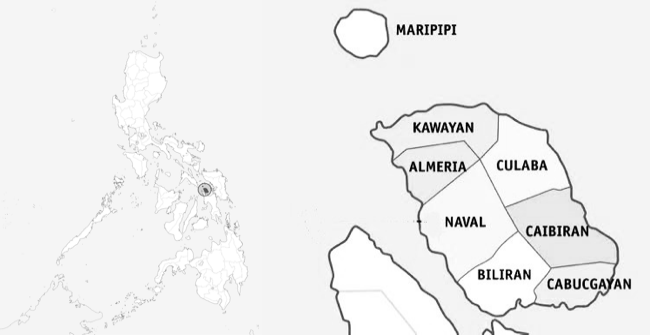
Map of Biliran Island and its municipalities *Source*: https://commons.wikimedia.org/wiki/user:Petriolo

The benthic foraminifera samples were collected within the coastal areas of eight municipalities on Biliran Island. Fig. 1 displays the map of Biliran Island and its corresponding municipalities where the samples were taken.

The findings of this study are consistent with previous studies that have focused on identifying benthic foraminifera species in coastal environments. For example, Ferraro and Hanauer (2014) conducted a study on the foraminifera distribution in the coastal areas of Italy and found that the distribution of foraminifera species was strongly correlated with environmental variables such as sediment type, water depth and salinity. El Albani *et al*. (2019) also found similar results in their study of the foraminifera distribution in the lagoon of Venice, Italy.

The identified benthic foraminifera found along the coastal area of the Biliran Province are the following: *Spirillina, Quinqueloculina, Marginopora* and *Sorites*. However, not all species are present in every municipality. Table 1 shows the distribution of benthic foraminifera in Biliran Island. Among the municipalities in Biliran Island, only in Naval coastal area where no species was found. In addition, the results of this study are also consistent with studies that have used benthic foraminifera as indicators of environmental change. For instance, Martinez-Colon *et al*. (2009) conducted a study on the impact of human activities on the foraminifera assemblages in the San Juan Bay, Puerto Rico. Their findings showed that the foraminifera assemblages in the impacted areas were different from those in the less impacted areas.

The samples were observed and compared to The World Foraminifera Database using a microscope to identify the specimen collected. Four species were identified based on the reference images as seen in Table 2.

The absence of benthic foraminifera in the coastal area of Naval is notable and requires further investigation. One possible explanation could be that the environmental conditions in this area are not suitable for the growth and survival of benthic foraminifera. This is consistent with the findings of Mojtahid *et al*. (2009), who found that the presence and abundance of benthic foraminifera were strongly correlated with environmental variables such as sediment type, water depth and salinity.

The identified benthic foraminifera in Biliran Island are classified based on their feeding mechanisms, which have implications on their ecological role in the marine ecosystem. For instance, some species engage in herbivory, such as *Quinqueloculina*, which are restricted to the euphotic zone and gather algae and bacteria with their reticulopodia. Other passive herbivores like *Textularia bocki* and *Quinqueloculina ungeriana* secrete glycosaminoglycans, which serve as a food source for bacteria and fungi, promoting their growth and providing a food source for the foraminifera (Gerlach 1978; Langer & Gehring 1993). Meanwhile, the passive suspension feeding mechanism is also present in the benthic foraminifera identified in Biliran Island, such as *Miliolinella* (Murray 2006). These species spread their reticulopodia in the water column and rely on water currents to bring food to them. They are sessile and epifaunal, rooted in soft sediment or attached to hard substrates. The positioning of their test erect and aperture above the substrate is crucial for their feeding strategy, as they require the water currents to bring food to them (Lipps 1983; Schönfeld 2002).

These different feeding mechanisms among the identified benthic foraminifera species in Biliran Island suggest that they play various ecological roles in the marine ecosystem. For instance, herbivorous foraminifera can affect the composition of the benthic community by consuming primary producers, while suspension feeders contribute to the transfer of organic matter from the water column to the benthic environment. However, more research is needed to fully understand the ecological implications of the identified benthic foraminifera species in Biliran Island.

Based on the study of Sumayao *et al*. (2021), there were species of benthic foraminifera present along the coastal water of Biliran Island. The benthic foraminifera species along the coastal environments of Biliran Island provides a record of the environment where they are found. Moreover, according to Tsujimoto *et al*. (2018), benthic foraminifera assemblages can also serve as indicators of the degree of human impact on the environment. The study of Martinez-Colon *et al*. (2009) supports this, stating that the distribution and abundance of benthic foraminifera in coastal sediments are sensitive indicators of environmental changes caused by human activities such as pollution and eutrophication. Similarly, Alve *et al*. (2009) emphasised the potential of benthic foraminifera as indicators of the impact of anthropogenic activities on the marine environment, particularly in areas of low-oxygen or hypoxic conditions. These findings suggest that the presence and absence of benthic foraminifera species in the coastal areas of Biliran Island could reflect the quality of the marine environment and the impact of human activities on it. Further studies on the relationship between benthic foraminifera assemblages and environmental factors such as water quality, pollution, and eutrophication can provide valuable information for the conservation and management of marine ecosystems.

The findings of this study provide valuable information on the distribution of benthic foraminifera species in the coastal areas of Biliran Island. Further studies could be conducted to investigate the environmental factors that influence the distribution and abundance of benthic foraminifera in this region, as well as to determine the potential use of benthic foraminifera as indicators of environmental change.

## CONCLUSION AND RECOMMENDATIONS

In conclusion, the study aimed to identify the benthic foraminifera species present along the coastal areas of Biliran Island. Through the methodology of setting up quadrats and collecting three samples per quadrant transect of 1 m × 1 m divided into nine squares, the researchers were able to identify four benthic foraminifera species: *Spirillina, Quinqueloculina, Marginopora* and *Sorites*. The distribution of these species in the different municipalities of Biliran Island was also determined. Further, the study discussed the feeding mechanisms of the identified species and their significance as bioindicators of water quality in coastal environments. The study recommended that future research on benthic foraminifera in the Philippines should focus on the potential use of these organisms as bioindicators of environmental change.

To improve the study, future researchers may consider expanding the scope of their study to include more areas in the Philippines and more variables that may affect the distribution and abundance of benthic foraminifera. They may also consider exploring the potential use of genetic markers to identify foraminifera species and studying their ecological roles in the marine ecosystem.

## Figures and Tables

**Table 1:** Benthic foraminifera presence in areas in Biliran Island.

Area	Naval	Almeria	Kawayan	Culaba	Caibiran	Cabucgayan	Biliran	Maripipi
Spirillina	−	+	+	+	−	−	+	+
Quinqueloculina	−	+	−	+	+	−	+	+
Marginopora	−	+	−	+	−	+	−	+
Sorites	−	+	−	+	−	+	−	+

*Note:* (+ presence; − absence)

**Table 2:** Collected benthic foraminifera samples present in the coastal areas in Biliran Island.

Taxonomy/Systematics		Foraminiferans observed using (4/0.1, 160/0.17) objective lens	Reference images from The World Foraminifera Database (https://marinespecies.org)
Kingdom:	Chromista	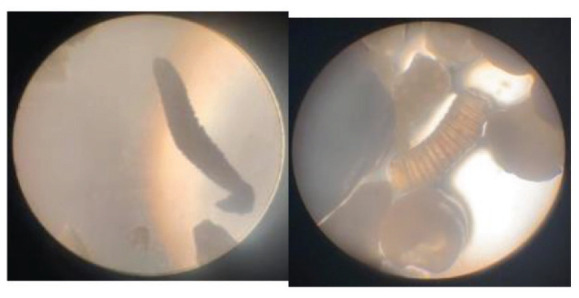	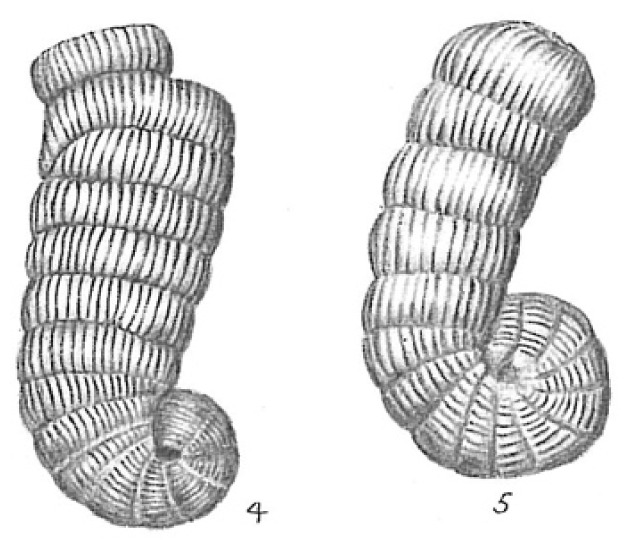
Subkingdom:	Harosa		
Infrakingdom:	Rhizaria		
Phylum:	Foraminifera		
Class:	Tubothalamea		
Order:	Spirillinida		
Suborder:	Spirillinina		
Family:	Spirillinidae		
Genus:	*Spirillina*		

Kingdom:	Chromista	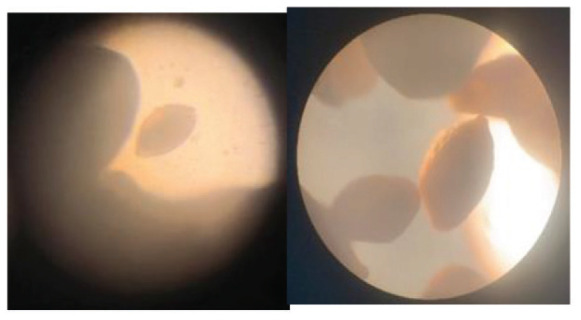	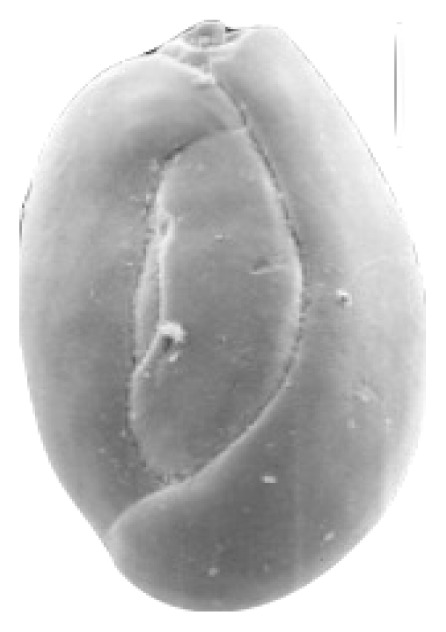
Subkingdom:	Harosa		
Infrakingdom:	Rhizaria		
Phylum:	Foraminifera		
Class:	Tubothalamea		
Order:	Miliolida		
Suborder:	Miliolida		
Family:	Hauerinidae		
Genus:	*Quinqueloculina*		
	*Quinqueloculina*		

Kingdom:	Chromista	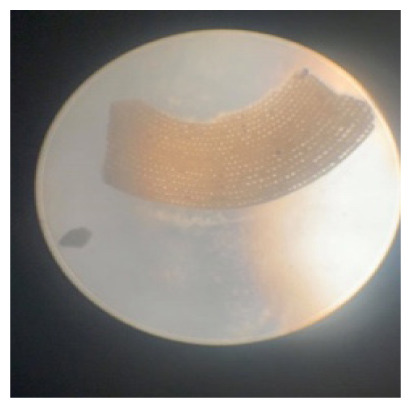	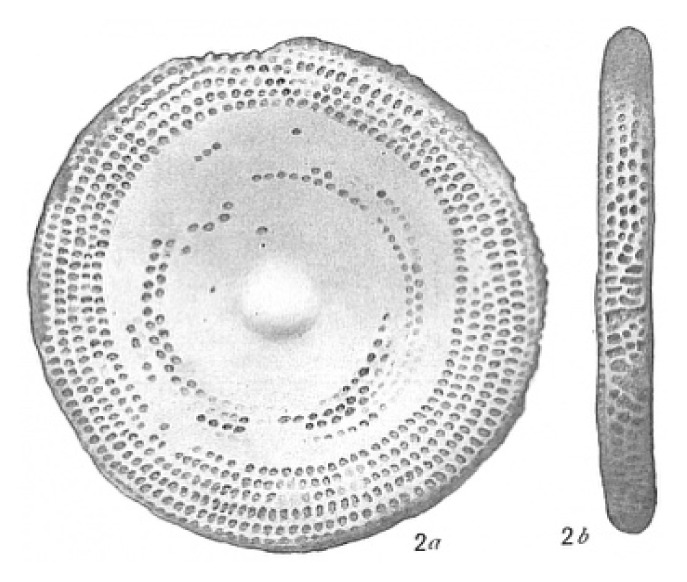
Subkingdom:	Harosa		
Infrakingdom:	Rhizaria		
Phylum:	Foraminifera		
Class:	Tubothalamea		
Order:	Miliolida		
Suborder:	Miliolina		
Family:	Soritidae		
Genus:	*Marginopora*		

Kingdom:	Chromista	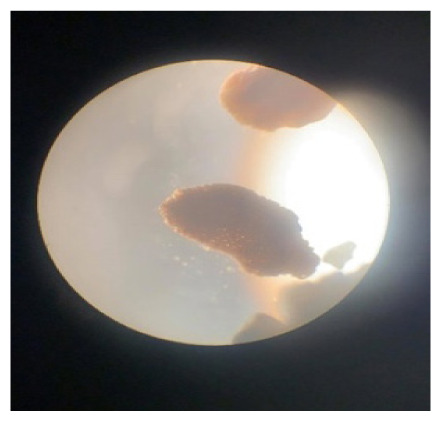	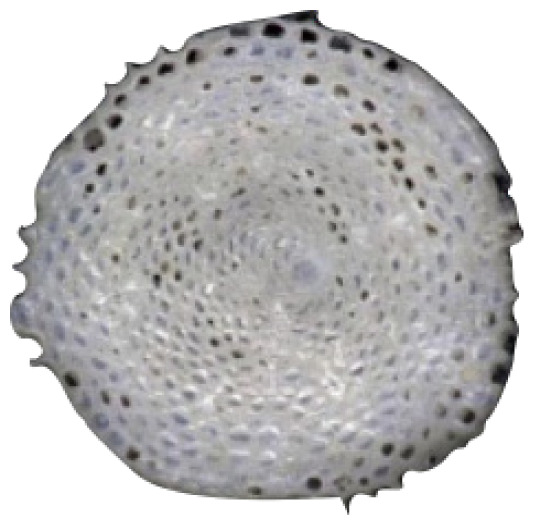
Subkingdom:	Harosa		
Infrakingdom:	Rhizaria		
Phylum:	Foraminifera		
Class:	Tubothalamea		
Order:	Miliolida		
Suborder:	Miliolina		
Family:	Soritidae		
Genus:	*Sorites*		
